# A diagnostic model based on routine blood examination for serious bacterial infections in neonates–a cross-sectional study

**DOI:** 10.1017/S0950268823001231

**Published:** 2023-07-31

**Authors:** Runqiang Liang, Ziyu Chen, Shumei Yang, Jie Yang, Zhu Wang, Xin Lin, Fang Xu

**Affiliations:** 1 National Key Clinical Specialty Construction Project/Department of Neonatology, Guangdong Women and Children Hospital, Guangzhou, China; 2 Guangdong Neonatal ICU Medical Quality Control Center, Guangzhou, China; 3 Department of Respiratory Medicine, Foshan Sanshui District People’s Hospital, Foshan, China; 4 Department of Pediatrics, Guangdong Women and Children Hospital, Guangzhou, China

**Keywords:** diagnostic model, neonates, routine blood examination, serious bacterial infections, MIMIC-III

## Abstract

Routine blood examination is an easy way to examine infectious diseases. This study is aimed to develop a model to diagnose serious bacterial infections (SBI) in ICU neonates based on routine blood parameters. This was a cross-sectional study, and data were extracted from the Medical Information Mart for Intensive Care III (MIMIC-III). SBI was defined as suffering from one of the following: pyelonephritis, bacteraemia, bacterial meningitis, sepsis, pneumonia, cellulitis, and osteomyelitis. Variables with statistical significance in the univariate logistic regression analysis and log systemic immune–inflammatory index (SII) were used to develop the model. The area under the curve (AUC) was calculated to assess the performance of the model. A total of 1,880 participants were finally included for analysis. Weight, haemoglobin, mean corpuscular volume, white blood cell, monocyte, premature delivery, and log SII were selected to develop the model. The developed model showed a good performance to diagnose SBI for ICU neonates, with an AUC of 0.812 (95% confidence interval (CI): 0.737–0.888). A nomogram was developed to make this model visualise. In conclusion, our model based on routine blood parameters performed well in the diagnosis of neonatal SBI, which may be helpful for clinicians to improve treatment recommendations.

## Introduction

Serious bacterial infection (SBI) is one of the leading causes of mortality in the ICU and accounts for 16.7% of mortality [1, 2]. Owing to the poorly developed immune system, the neonatal population in the ICU is considered the high-risk group to suffer from infections [3, 4]. Early identification of SBI might be contributed to favourable treatment outcomes; however, there are few obvious clinical symptoms and signs to indicate SBI in neonates [5]. Therefore, it is a challenging task for the early diagnosis of neonatal SBI in the ICU.

Routine blood examination is an easy and common way used to examine infectious diseases [6]. Some studies have reported a high diagnostic accuracy of routine blood parameters in infectious diseases [7, 8]. Mentis et al. confirmed that routine blood parameters can be effectively used for the diagnosis of meningitis [7]. Tschoellitsch et al. [8] found that machine learning methods based on routine blood examination can reliably diagnose pneumonia. In addition, combined routine blood parameters, such as neutrophil-to-lymphocyte ratio and platelet-to-lymphocyte ratio, have been used as markers for the diagnosis of infectious diseases [9, 10].

In the neonates admitted to ICU, the diagnostic value of routine blood parameters in SBI was also reported [11, 12]. Guo et al. [11] reported that red cell distribution width (RDW) was an important factor to diagnose SBI in neonates in the ICU. Moreover, neutrophil and white blood cell counts (WBC) were identified as effective markers for the early diagnosis of neonatal SBI in the ICU [12]. Although these markers have been reported, the diagnostic value of routine blood parameters for SBI in neonates admitting to the ICU needed to further explore.

In this study, we aim to develop a diagnostic model for neonatal SBI based on routine blood parameters using the data from the Medical Information Mart for Intensive Care III (MIMIC-III) database.

## Methods

This study on the diagnostic model was reported according to the Transparent Reporting of a multivariable prediction model for Individual Prognosis or Diagnosis (TRIPOD) statement [13].

### Study design and data source

This was a cross-sectional study, and data were extracted from MIMIC-III. MIMIC-III was a large and single-centre database, comprising clinical data on patients admitted to the ICU in the Beth Israel Deaconess Medical Center in Boston, Massachusetts [14]. The clinical data were collected based on laboratory measurements, medications, vital signs, length of hospital stay, survival, and so forth. This database supported applications including academic research, higher education coursework, and quality improvement initiatives. Our study was not required to sign the informed consent because MIMIC-III was a publicly available database.

### Participants

Neonates (<28 days) admitted to the neonatal intensive care unit (NICU) ≥ 24 hours were included in this study, and neonates of missing data on routine blood examination were excluded.

### Variables for diagnostic model

#### Candidate variables

Data were extracted within 24 hours after neonates were admitted to the NICU based on demographic characteristics (gender, weight, race, premature delivery, and cesarean section delivery) and routine blood examination (monocyte, mean corpuscular volume (MCV), haemoglobin (HGB), RDW, WBC count, lymphocyte count, neutrophil count, and platelet count)], and systemic immune–inflammatory index (SII). SII was a new marker of inflammation, which was calculated by the combination of platelet, neutrophil, and lymphocyte counts (that is platelet counts × neutrophil counts/lymphocyte counts) and reflected the balance between inflammatory and immune statuses [15]. Premature birth was categorised into extremely preterm (< 28 weeks), very preterm (28 to 32 weeks), moderate preterm (32 to 34 weeks), and late preterm (34 to 37 weeks) [16].

#### Dependent variable

The dependent variable was SBI. According to the previously reported study [17], SBI was defined as suffering from one of the following diseases: pyelonephritis, bacteraemia, bacterial meningitis, sepsis, pneumonia, cellulitis, and osteomyelitis. These diseases were identified from the medical record.

### Development and internal validation of the model

This diagnostic model was developed using the logistic regression method. Participants were divided into training set and testing set in a ratio of 7:3. Variables with a statistical significance in the univariate logistic regression analysis were selected to develop the model. Considering that SII was reported as an effective marker to diagnose SBI in age groups where the immune system was immature [15], SII was also included in the model. Quantile–quantile (Q-Q) plot was used to graphically assess whether the dataset was in a normal distribution [18], and SII in skew distribution was normalised using logarithm method (log SII) (Supplementary Figure S1A,B).

The performance of this model was evaluated by calculating the area under the curve (AUC), accuracy, specificity, sensitivity, positive predictive value, and negative predictive value (NPV). Calibration was evaluated using a visual calibration plot. This diagnostic model was internally validated using 10-fold cross-validation. The discrimination capacity in each of the 10-fold cross-validation subsamples was calculated, and the mean cross-validated AUC was reported. The diagnostic result of this model was visualised using a nomogram.

### Statistical analysis

Continuous data in normal distribution were described as mean ± standard deviation (mean ± SD), and differences between the two groups were compared using an independent sample t-test. Continuous data in skew distribution were described as a median and interquartile range [M (Q1, Q3)], and differences between the two groups were compared using Mann–Whitney U rank-sum test. The categorical data were described as number and percentage [N (%)], and differences between the two groups were compared using a chi-square test. Missing data were processed by deletion. Univariate logistic regression analysis was used to select factors to develop the diagnostic model, and the results were reported as odds ratio (OR) and 95% confidence intervals (95% CI). Statistical analysis was performed using SAS 9.4 (SAS Institute Inc., Cary, NC) and R (version 4.0.3, Institute for Statistics and Mathematics, Vienna, Austria), and the statistical significance was displayed by *P* < 0.05.

## Results

### Participants

**Figure 1.**
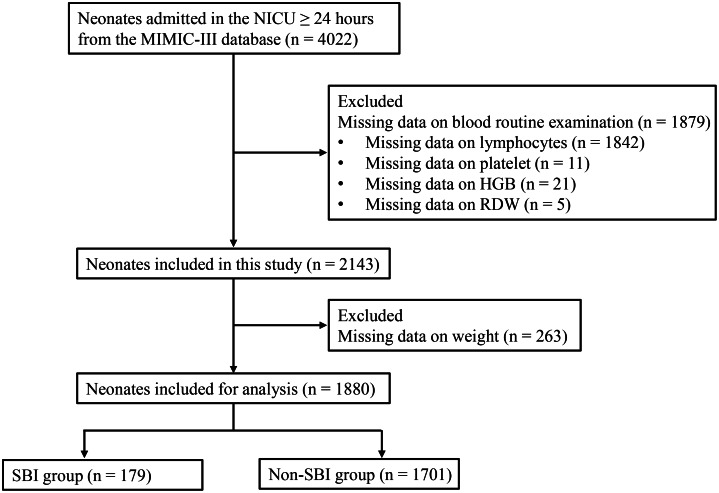
The flowchart of participant selection.

A total of 4,022 neonates admitted to the NICU ≥24 hours were extracted from the MIMIC-III database. Of these, 1,879 neonates missing data on routine blood examination were excluded (1,842 neonates missing data on lymphocytes, 11 neonates missing data on platelets, 21 neonates missing data on HGB, and 5 neonates missing data on RDW). Further, 263 neonates missing data on weight were excluded. Finally, 1,880 neonates were included for analysis, with 179 neonates in the SBI group and 1,701 neonates in the non-SBI group (Figure 1). There was 55.90% of males in the included participants. A significant difference was observed in weight, premature delivery, monocyte, MCV, HGB, WBC, and platelets between the SBI group and non-SBI group (Table 1).

**Table 1.** Characteristics of neonates with and without SBI

Variables	Total (*n* = 1,880)	Non-SBI (*n* = 1,701)	SBI (*n* = 179)	*P*
Gender, *n* (%)				0.643
Female	829 (44.10)	753 (44.27)	76 (42.46)	
Male	1,051 (55.90)	948 (55.73)	103 (57.54)	
Weight, kg, M (Q_1_, Q_3_)	1.98 (1.24, 2.68)	2.06 (1.36, 2.72)	0.92 (0.68, 1.39)	<0.001
Race, *n* (%)				0.093
Asian	115 (6.12)	108 (6.35)	7 (3.91)	
Black	203 (10.80)	176 (10.35)	27 (15.08)	
White	1,183 (62.93)	1,079 (63.43)	104 (58.10)	
Other	379 (20.16)	338 (19.87)	41 (22.91)	
Premature delivery, *n* (%)				<0.001
No	275 (14.63)	263 (15.46)	12 (6.70)	
Extremely preterm	262 (13.94)	158 (9.29)	104 (58.10)	
Very preterm	685 (36.44)	642 (37.74)	43 (24.02)	
Moderate preterm	423 (22.50)	414 (24.34)	9 (5.03)	
Late preterm	235 (12.50)	224 (13.17)	11 (6.15)	
Caesarean section delivery, *n* (%)				0.157
No	603 (32.07)	554 (32.57)	49 (27.37)	
Yes	1,277 (67.93)	1,147 (67.43)	130 (72.63)	
Monocyte, 10^3^/uL, M (Q_1_, Q_3_)	7.00 (4.00, 9.00)	7.00 (4.00, 9.00)	7.00 (5.00, 11.00)	0.002
MCV, fL, Mean ± SD	110.32 ± 7.89	109.85 ± 7.61	114.77 ± 9.02	<0.001
HGB, g/dL, Mean ± SD	16.12 ± 2.32	16.23 ± 2.30	15.06 ± 2.27	<0.001
RDW, %, Mean ± SD	17.26 ± 1.45	17.25 ± 1.37	17.35 ± 2.07	0.514
WBC, K/uL, M (Q_1_, Q_3_)	11.40 (8.00, 15.85)	7.60 (5.30, 13.00)	11.70 (8.50, 16.10)	<0.001
Lymphocytes, %, M (Q_1_, Q_3_)	46.00 (29.00, 62.00)	46.00 (29.00, 62.00)	45.00 (31.00, 62.00)	0.779
Neutrophil, %, M (Q_1_, Q_3_)	39.00 (25.00, 58.00)	39.00 (25.00, 58.00)	41.00 (27.00, 57.00)	0.468
Platelet, K/uL, M (Q_1_, Q_3_)	257.00 (209.00, 314.00)	260.00 (214.00, 316.00)	228.00 (176.00, 287.00)	<0.001
Log SII, Mean ± SD	5.36 ± 1.14	5.36 ± 1.14	5.34 ± 1.10	0.830

Abbreviations: HGB, haemoglobin; MCV, mean corpuscular volume; RDW, red blood cell distribution width; SBI, serious bacterial infections; SII, systemic immune–inflammation index; WBC, white blood cell.

### Model development and performance

**Figure 2.**
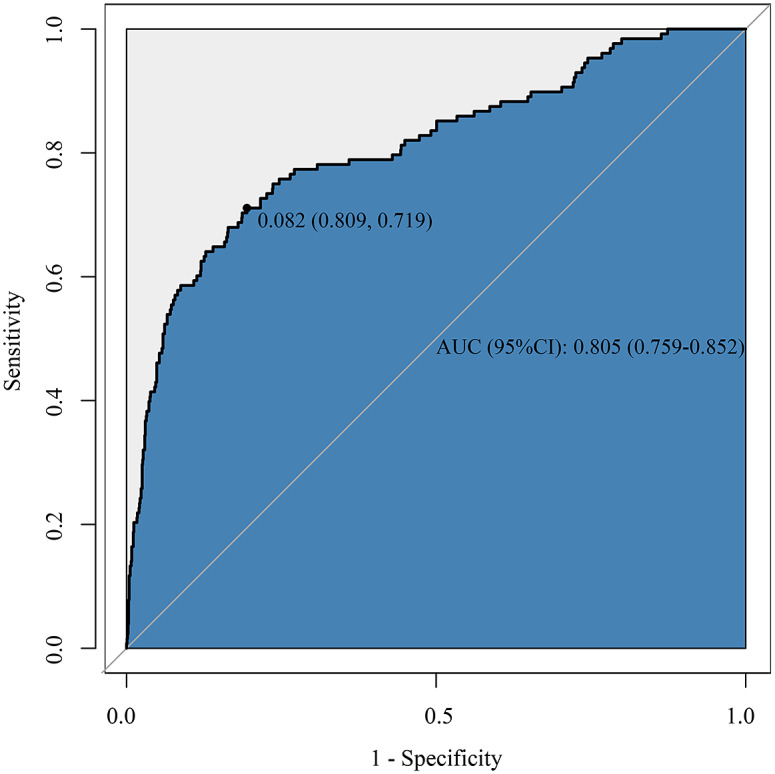
ROC curve of the training set.

**Figure 3.**
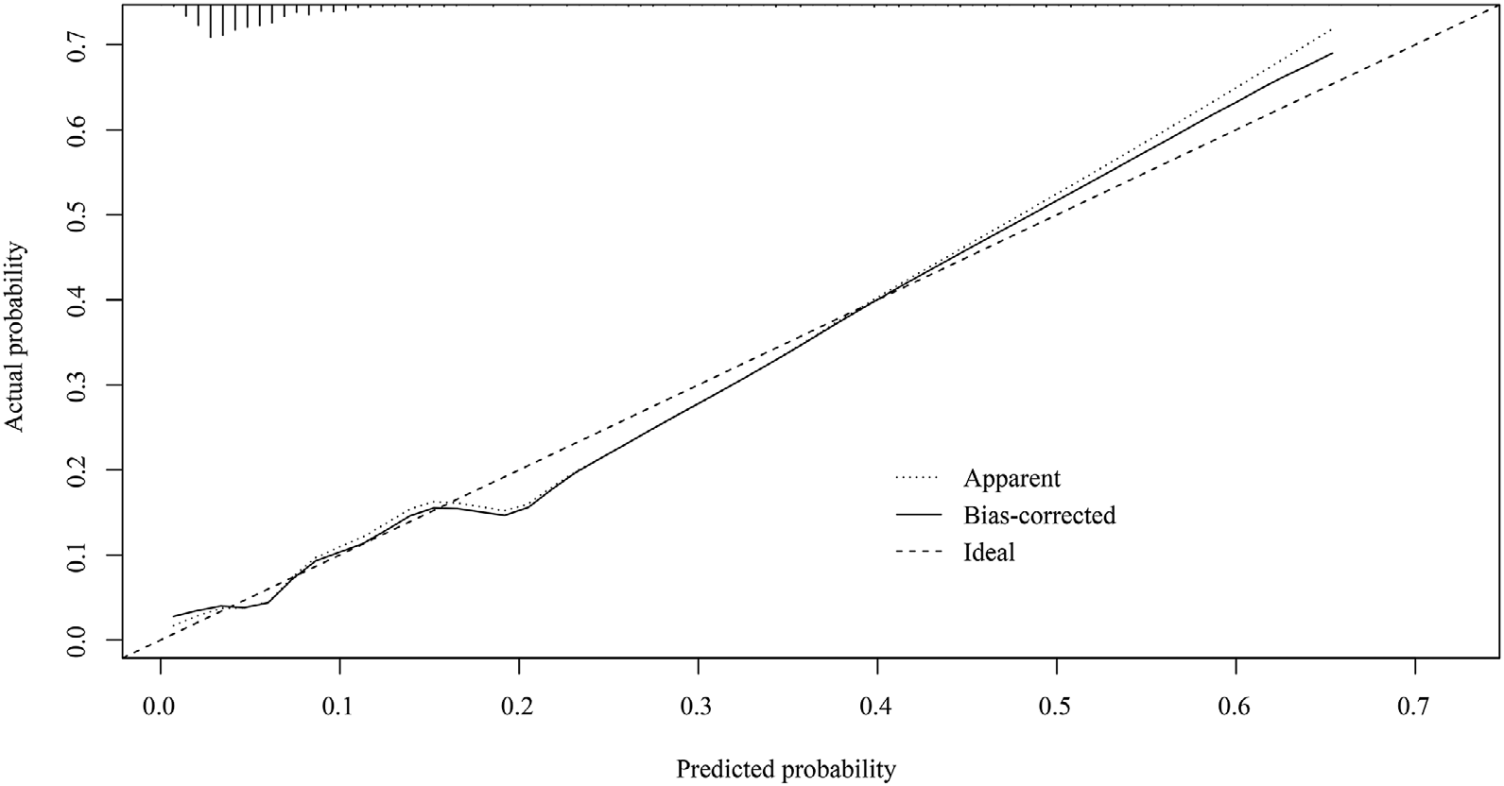
Calibration plot of the diagnostic model for the probability of SBI in ICU neonates.

Supplementary Table S1 shows that weight (OR = 0.43, 95%CI: 0.34–0.53), premature delivery (extremely preterm: OR = 27.34, 95%CI: 12.72–58.74; very preterm: OR = 2.74, 95%CI: 1.25–6.04), monocyte (OR = 1.33, 95%CI: 1.13–1.57), MCV (OR = 1.09, 95%CI: 1.06–1.11), HGB (OR = 0.64, 95%CI: 0.53–0.77), and WBC (OR = 1.83, 95%CI: 1.20–2.79) were associated with SBI. Therefore, the diagnostic model was developed based on weight, HGB, MCV, WBC, monocyte, premature delivery, and log SII (Supplementary Table S2). The AUC of this model was 0.805 (95%CI: 0.759–0.852) (Table 2). At a cut-off value of 0.082, the maximum specificity and sensitivity were 0.809 (95%CI: 0.787–0.831) and 0.719 (95%CI: 0.641–0.797), respectively (Figure 2). The calibration plot demonstrated that the predictive probability of SBI fitted well with the actual probability (Figure 3), indicating good calibration of our model.

**Table 2.** Performance of the diagnostic model for neonatal SBI based on routine blood examination

Variables	AUC (95%CI)	Accuracy (95%CI)	Specificity (95%CI)	Sensitivity (95%CI)	PPV (95%CI)	NPV (95%CI)
Training set	0.805 (0.759–0.852)	0.800 (0.778–0.821)	0.809 (0.787–0.831)	0.719 (0.641–0.797)	0.288 (0.239–0.338)	0.964 (0.952–0.975)
Testing set	0.812 (0.737–0.888)	0.762 (0.725–0.797)	0.762 (0.725–0.799)	0.765 (0.648–0.881)	0.242 (0.176–0.308)	0.970 (0.954–0.987)

Abbreviations: AUC, the area under the curve; CI, confidence interval; NPV, negative predictive value; PPV, positive predictive value; SBI, serious bacterial infections.

The performance of this model was verified by internal validation using the testing set, and the AUC was 0.812 (95%CI: 0.737–0.888) (Table 2), indicating a good discrimination capacity of this diagnostic model. In addition, the 10-fold cross-validation in the full dataset showed that the mean AUC within the 10 folds was 0.818 (95%CI: 0.814–0.821) (Supplementary Table S3).

### Nomogram

**Figure 4.**
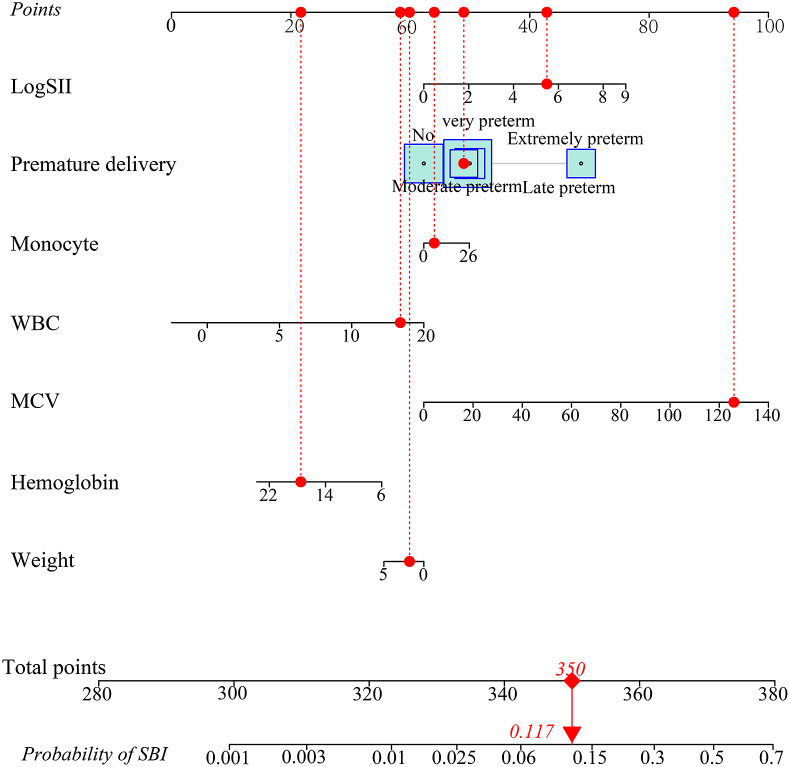
Nomogram for the diagnosis of SBI in ICU neonates.

A diagnostic nomogram was developed based on the factors in the diagnostic model. A total score was obtained by summing up the single score of each factor and used to estimate the probability of SBI. For example, a patient with a weight of 1.78 kg, moderate preterm, 6 * 10^3^/uL of monocyte, 126 fL of MCV, 17.5 g/dL of HGB, 15.8 K/uL of WBC, and log SII in 5.49 had a total score of 350 points, with 11.7% of probability to diagnose as SBI (Figure 4).

## Discussion

Neonates are the high-risk group to suffer from SBI due to their immature immune system, which raises the risk of disability and death in the world [1, 3]. Some studies have reported the potential diagnostic utility of routine blood parameters in infectious diseases [7, 8]. In this study, we developed a diagnostic model based on routine blood parameters for SBI in neonates admitted to the ICU. Weight, HGB, MCV, WBC, monocyte, premature delivery, and log SII were used to develop the model. Results showed that the model performed well in the diagnosis of SBI for neonates admitted to the ICU. The model was visualised using a nomogram, which may be convenient to use and be helpful for clinicians to improve treatment recommendations.

SII was an integrated parameter based on platelet, neutrophil, and lymphocyte counts, which gained extraordinary popularity as a routine blood-based marker in the clinic since Hu et al. introduced it in 2014 [19]. As an integrated indicator, SII might be better to reflect the balance between host inflammatory and immune status [15, 19]. Previous studies have reported the clinical values of SII in cancers, coronavirus disease 2019 (COVID-19), and urinary tract infections [20–23]. SII also showed usability in age groups where the immune system was immature [15]. Güngör et al. [15] found that SII was a predictor for urinary tract infections in infants. In our study, SII was identified as an important factor to diagnose SBI in neonates. Although the diagnostic performance of SII might be dissimilar in different age groups, diagnosed diseases, and severity of diseases, its advantages in a straightforward calculation from hemogram results and not requiring additional costs and further blood collection cannot be ignored [15].

WBC, HGB, MCV, and monocyte were also used to develop the model. WBC is the main immune cell in human bodies and has been reported to diagnose bacterial infections and assess the severity of diseases effectively [24, 25]. In the clinic, WBC is needed to combine with other hematological indexes to diagnose infectious diseases because its level could be regulated by some inflammatory responses and infections [25]. HGB was also one of the hematological indexes and has been reported to be associated with the prognosis of SBI patients [26]. The association between monocyte and bacterial infection has been reported, and mean monocyte volume increased in postsurgical bacterial infection [27]. In addition, MCV was a measure of the mean size of erythrocytes and has long been a useful index for diagnosing infectious diseases [28, 29]. The mechanism underlying the association between MCV and SBI was unclear. One potential explanation was that there was an association between a high MCV and malnutrition, which caused a decrease in immunologic function, thereby increasing the susceptibility to infections [30, 31]. In addition, evidence has shown that premature delivery and weight were also influencing factors for the diagnosis of SBI [32, 33]. Our model developed based on log SII, WBC, HGB, MCV, monocyte, weight, and premature delivery showed a good performance to diagnose SBI, with an AUC of 0.812.

Also, we developed a nomogram based on the diagnostic model. Nomogram was a visual tool that could simplify a large number of complex factors into a single simple numerical estimation model to determine the probability of events [34]. The nomogram in this study may be convenient for clinicians to use. Using the nomogram, clinicians could make individualised predictions of SBI probability for each patient. Our model showed high performance for the prediction of negative results due to the high value of NPV and specificity. For individuals who were predicted with positive results, more examinations should be performed to further verify the diagnostic results. Our model was developed based on routine blood parameters, which were easy to obtain using a routine analyser [6]. If an algorithm was embedded within a blood analyser to report a probability of SBI in the lab results, it might be helpful for clinicians to determine to start or stop the use of antibiotics. In future, more effort is still needed to optimise this diagnostic model to make it be practicable in the clinic.

There are several limitations to this study. First, our data are extracted from the MIMIC-III database, and data missing is inevitable. Participants with missing data are excluded from our study, which may cause a decrease in the sample size, thereby reducing the statistical power of the model. Second, SBI is defined based on medical records rather than the results of bacterial culture, which may exist reporting bias. Third, our model is short of external validation and validation in non-ICU settings. Future studies are needed to verify the performance of our model in external patients and non-ICU populations.

## Conclusion

We developed a model based on routine blood examination to diagnose SBI in neonates admitted to the ICU, and this model showed a good performance. Nomogram was developed to make the model convenient to use in the clinic. By applying this model, clinicians could predict each individual’s probability of SBI and improve treatment recommendations for a high-risk population.

## Supporting information

Liang et al. supplementary material 1 Liang et al. supplementary material 1

Liang et al. supplementary material 2 Liang et al. supplementary material 2

Liang et al. supplementary material 3 Liang et al. supplementary material 3

## Data Availability

The datasets used and/or analysed during the current study are available from the corresponding author on reasonable request.

## Supplementary material

The supplementary material for this article can be found at https://doi.org/10.1017/S0950268823001231.
