# Supplementary material for: A diagnostic model based on routine blood examination for serious bacterial infections in neonates–a cross-sectional study
Source: Epidemiol Infect. 2023 Jul 31;151:e137. doi: 10.1017/S0950268823001231 (PMC10540195; doi:10.1017/S0950268823001231)
Supplement: Liang et al. supplementary material 1 [file S0950268823001231sup001.docx]

**Supplementary table S1 Screening of variables for the development of diagnostic model**

| **Variables** | **OR (95%CI)** | ***P*** |
| --- | --- | --- |
| Gender |  |  |
| Female | Ref |  |
| Male | 1.11 (0.77-1.60) | 0.587 |
| Weight | 0.43 (0.34-0.53) | < 0.001 |
| Race |  |  |
| Asian | Ref |  |
| Black | 2.65 (0.95-7.40) | 0.062 |
| White | 1.60 (0.63-4.06) | 0.327 |
| Other | 2.01 (0.76-5.34) | 0.162 |
| Premature delivery |  |  |
| No | Ref |  |
| Extremely preterm | 27.34 (12.72-58.74) | < 0.001 |
| Very preterm | 2.74 (1.25-6.04) | 0.012 |
| Moderate preterm | 1.76 (0.63-4.95) | 0.283 |
| Late preterm | 2.16 (0.84-5.58) | 0.111 |
| Cesarean section delivery |  |  |
| No | Ref |  |
| Yes | 1.21 (0.81-1.80) | 0.359 |
| Monocyte | 1.33 (1.13-1.57) | < 0.001 |
| MCV | 1.09 (1.06-1.11) | < 0.001 |
| HGB | 0.64 (0.53-0.77) | < 0.001 |
| RDW | 1.09 (0.92-1.29) | 0.344 |
| WBC | 1.83 (1.20-2.79) | 0.005 |

Abbreviation: MCV, mean corpuscular volume; HGB, hemoglobin; RDW, red blood cell distribution width; WBC, white blood cell.

**Supplementary table S2 Variables used to develop the diagnostic model**

|  | Estimate | Std. Error | z value | Pr(>\|z\|) |
| --- | --- | --- | --- | --- |
| (Intercept) | -7.2424 | 2.0292 | -3.5691 | < 0.001 |
| Weight | -0.1292 | 0.159 | -0.8126 | 0.4164 |
| HGB | -0.1135 | 0.0496 | -2.2852 | 0.0223 |
| MCV | 0.0398 | 0.0153 | 2.6045 | 0.0092 |
| WBC | 0.0583 | 0.0205 | 2.8473 | 0.0044 |
| Monocyte | 0.0283 | 0.0234 | 1.2104 | 0.2261 |
| Premature delivery (extremely preterm) | 2.5424 | 0.4697 | 5.4127 | < 0.001 |
| Premature delivery (very preterm) | 0.7081 | 0.4299 | 1.6473 | 0.0995 |
| Premature delivery (moderate preterm) | 0.6468 | 0.5341 | 1.211 | 0.2259 |
| Premature delivery (late preterm) | 0.7428 | 0.4883 | 1.5212 | 0.1282 |
| Log SII | 0.3624 | 0.1229 | 2.9493 | 0.0032 |

Abbreviation: HGB, hemoglobin; MCV, mean corpuscular volume; WBC, white blood cell; SII, systemic immune-inflammation index.

**Supplementary table S3 Internal validation for the performance of the diagnostic model using 10-fold cross-validation**

| **Variables** | **AUC (95%CI)** | **Accuracy (95%CI)** | **Specificity (95%CI)** | **Sensitivity (95%CI)** | **PPV (95%CI)** | **NPV (95%CI)** |
| --- | --- | --- | --- | --- | --- | --- |
| Mean 10-fold cross validation | 0.818 (0.814-0.821) | 0.810 (0.803-0.816) | 0.821 (0.813-0.829) | 0.697 (0.687-0.707) | 0.292 (0.284-0.299) | 0.963 (0.962-0.964) |
| Fold 1 | 0.810 (0.770-0.849) | 0.795 (0.775-0.814) | 0.805 (0.785-0.825) | 0.696 (0.625-0.767) | 0.273 (0.230-0.316) | 0.962 (0.951-0.972) |
| Fold 2 | 0.808 (0.769-0.848) | 0.793 (0.772-0.812) | 0.802 (0.782-0.822) | 0.702 (0.631-0.773) | 0.272 (0.229-0.314) | 0.962 (0.952-0.973) |
| Fold 3 | 0.819 (0.781-0.857) | 0.801 (0.782-0.820) | 0.812 (0.792-0.831) | 0.702 (0.631-0.773) | 0.282 (0.238-0.326) | 0.963 (0.952-0.973) |
| Fold 4 | 0.818 (0.779-0.857) | 0.822 (0.802-0.839) | 0.838 (0.820-0.856) | 0.665 (0.592-0.738) | 0.301 (0.254-0.349) | 0.960 (0.949-0.970) |
| Fold 5 | 0.816 (0.777-0.855) | 0.819 (0.799-0.837) | 0.831 (0.813-0.850) | 0.696 (0.625-0.767) | 0.303 (0.256-0.350) | 0.963 (0.953-0.973) |
| Fold 6 | 0.823 (0.785-0.860) | 0.805 (0.785-0.824) | 0.815 (0.796-0.835) | 0.708 (0.638-0.778) | 0.287 (0.243-0.332) | 0.964 (0.954-0.974) |
| Fold 7 | 0.827 (0.791-0.864) | 0.817 (0.798-0.835) | 0.830 (0.811-0.849) | 0.689 (0.618-0.761) | 0.299 (0.253-0.346) | 0.962 (0.952-0.972) |
| Fold 8 | 0.821 (0.783-0.859) | 0.823 (0.804-0.841) | 0.837 (0.819-0.856) | 0.685 (0.614-0.757) | 0.308 (0.261-0.356) | 0.962 (0.951-0.972) |
| Fold 9 | 0.822 (0.784-0.859) | 0.808 (0.788-0.826) | 0.816 (0.797-0.836) | 0.727 (0.658-0.796) | 0.294 (0.249-0.339) | 0.966 (0.956-0.976) |
| Fold 10 | 0.813 (0.773-0.852) | 0.814 (0.795-0.833) | 0.827 (0.808-0.846) | 0.696 (0.625-0.767) | 0.297 (0.251-0.343) | 0.963 (0.953-0.973) |

AUC, the area under the curve; PPV, positive predictive value; NPV, negative predictive value; CI, confidence interval.
