# Supplementary figures and images for: A diagnostic model based on routine blood examination for serious bacterial infections in neonates–a cross-sectional study
Source: Epidemiol Infect. 2023 Jul 31;151:e137. doi: 10.1017/S0950268823001231 (PMC10540195; doi:10.1017/S0950268823001231)

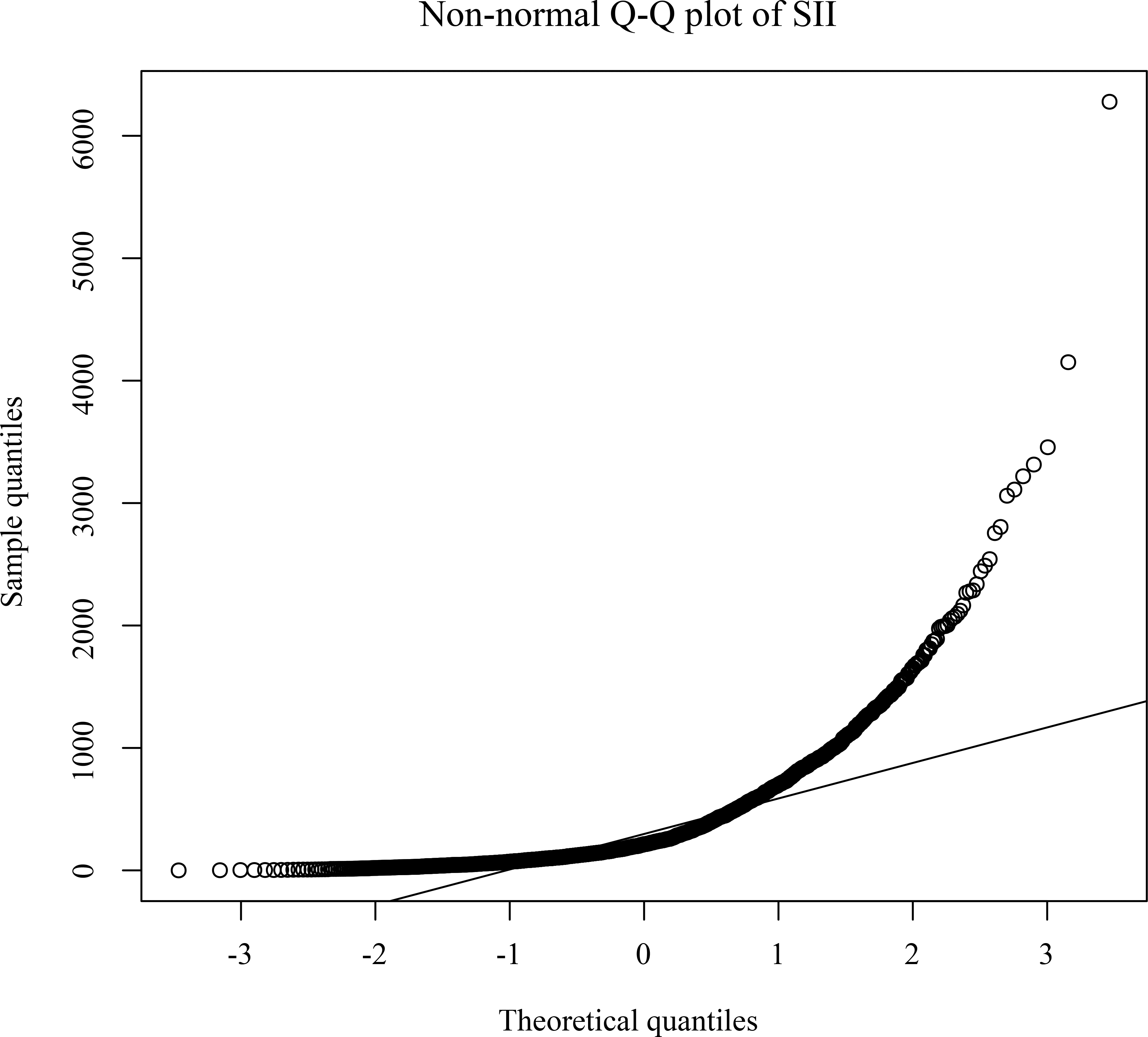

Supplement: Liang et al. supplementary material 2 [file S0950268823001231sup002.png]

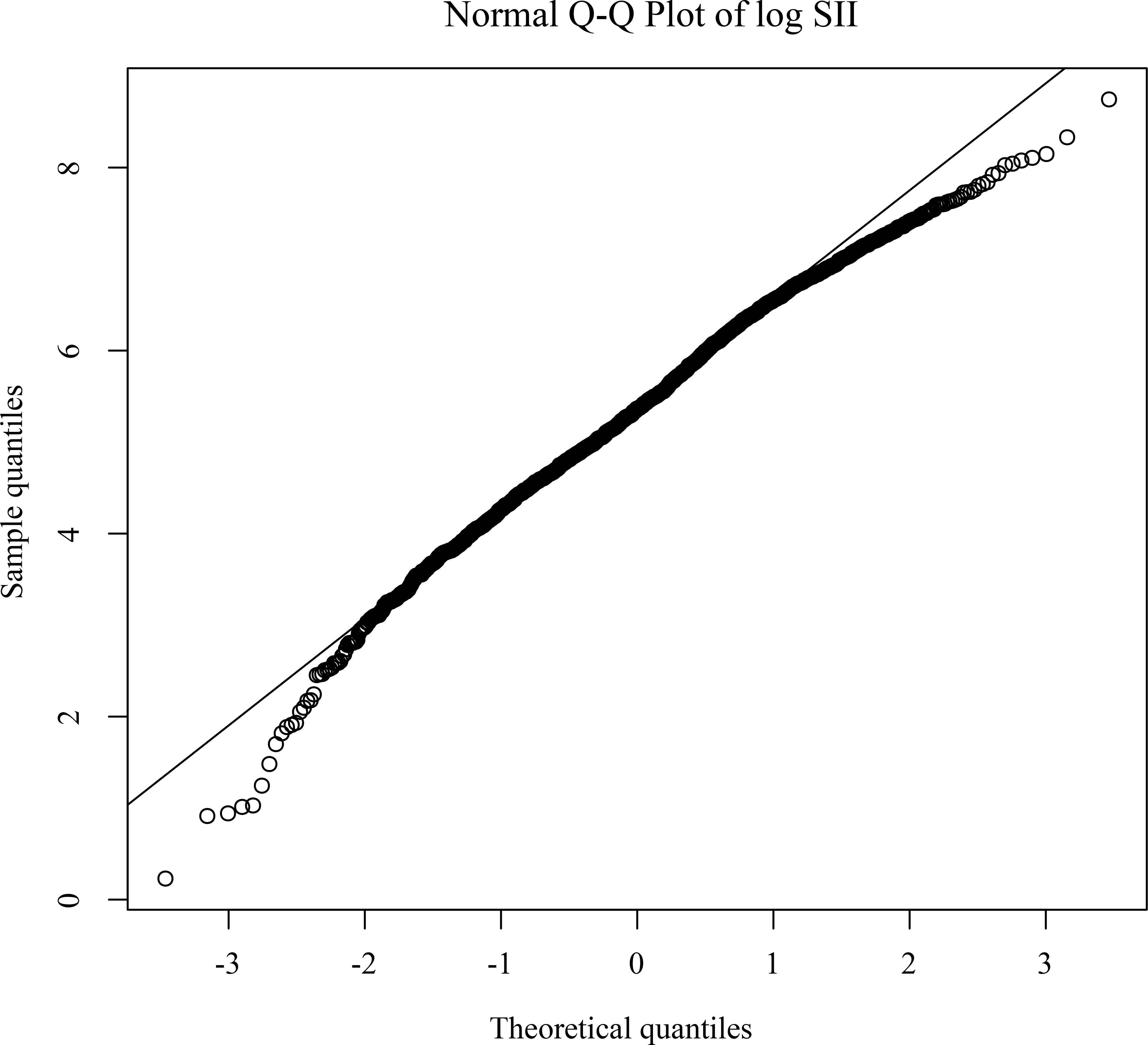

Supplement: Liang et al. supplementary material 3 [file S0950268823001231sup003.png]
